# Role of p40*^phox^* in host defense against *Citrobacter* *rodentium* infection

**DOI:** 10.1002/2211-5463.13155

**Published:** 2021-04-09

**Authors:** Yanyun Yan, Yali Li, Meili Lv, Weifen Li, Hai Ning Shi

**Affiliations:** ^1^ Hunan Provincial Key Laboratory of Animal Intestinal Function and Regulation College of Life Sciences Hunan Normal University Changsha China; ^2^ Sichuan University Chengdu China; ^3^ Zhejiang University Hangzhou China; ^4^ Mucosal Immunology and Biology Research Center Harvard Medical School Massachusetts General Hospital Charlestown MA USA

**Keywords:** *C. rodentium*, colonic inflammation, IBD, p40*^phox^*

## Abstract

NADPH oxidase (NOX) is a membrane‐bound enzyme complex that generates reactive oxygen species (ROS). Mutations in NOX subunit genes have been implicated in the pathogenesis of inflammatory bowel disease (IBD), indicating a crucial role for ROS in regulating host immune responses. In this study, we utilize genetically deficient mice to investigate whether defects in p40*^phox^*, one subunit of NOX, impair host immune response in the intestine and aggravate disease in an infection‐based (*Citrobacter rodentium*) model of colitis. We show that p40*^phox^* deficiency does not increase susceptibility of mice to *C. rodentium* infection, as no differences in body weight loss, bacterial clearance, colonic pathology, cytokine production, or immune cell recruitment were observed between p40*^phox^* ^−/−^ and wild‐type mice. Interestingly, higher IL‐10 levels were observed in the supernatants of MLN cells and splenocytes isolated from infected p40*^phox^*‐deficient mice. Further, a higher expression level of inducible nitric oxide synthase (iNOS) was also noted in mice lacking p40*^phox^*. In contrast to wild‐type mice, p40*^phox^* ^−/−^ mice exhibited greater NO production after LPS or bacterial antigen re‐stimulation. These results suggest that p40*^phox^* ^−/−^ mice do not develop worsened colitis. While the precise mechanisms are unclear, it may involve the observed alteration in cytokine responses and enhancement in levels of iNOS and NO.

## Graphical abstract

We investigated the role of p40*^phox^* in *Citrobacter rodentium* infection. Although p40*^phox^* ^−/−^ mice did not develop worsened colitis, higher IL‐10 levels were observed in MLN cells and splenocytes isolated from infected p40*^phox^* ^−/−^ mice compared with wild‐type mice. Further, higher expression level of iNOS and greater NO production was noted in p40*^phox^* ^−/−^ lymphocytes after LPS or bacterial antigen re‐stimulation.

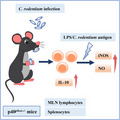

**Abbreviations**
B6: C57BL/6
*C. rodentium*: *Citrobacter rodentium*
C‐Ag: *C. rodentium* antigen
CGD: chronic granulomatous disease
DSS: dextran sulfate sodium
IBD: inflammatory bowel disease
IFN‐γ: interferon gamma
IL: interleukin
iNOS: inducible nitric oxide synthase
KO: knockout
MGH: Massachusetts General Hospital
MLN: mesenteric lymph node
NO: nitric oxide
NOX: NADPH oxidase
p40*^phox^*^−^^/^^−^: p40*^phox^* knockout
ROS: reactive oxygen species
TNF‐α: tumor necrosis factor alpha

Inflammatory bowel disease is a chronic inflammatory disorder of gastrointestinal tract, mainly mediated by genetic, immune, microbiota, and environmental factors [1, 2]. Ulcerative colitis and Crohn’s disease are the principal types of IBD [3], which are both conditions characterized by exaggerated immune responses directed against the enteric bacteria or their components [4]. As there is no known effective cure, current treatments primarily aim at alleviating intestinal inflammation or ameliorating symptoms [5]. Thus, there is an urgent need to identify the contributing factors in the pathogenesis of IBD, which will undoubtedly aid in developing more effective treatment strategies.

Currently, increasing number of susceptibility genes has been identified as contributing to IBD pathogenesis [6, 7]. Association studies have linked NADPH oxidase complex that mediates oxidative burst with the development of IBD [8]. Phagocyte NADPH oxidase (NOX), composed of six subunits: gp91*^phox^*, p22*^phox^*, p47*^phox^*, p67*^phox^*, p40*^phox^*, and Rac2, plays a key role in microbial killing through the generation of reactive oxygen species (ROS) [9]. Deficiency in ROS production is usually associated with enhanced susceptibility to infection and chronic granulomatous disease (CGD) [10, 11, 12]. As high as 40% of individuals with CGD develop Crohn’s disease‐like intestinal inflammation [13], highlighting ROS and innate immunity as critical components in intestinal homeostasis. Previous studies have revealed that Rac2 deficiency could result in exacerbated and protracted colitis in response to *C. rodentium* infection [5] while mice deficient in gp91*^phox^* or p47*^phox^* displayed no overt differences during acute dextran sulfate sodium (DSS) colitis [14, 15]. Moreover, Pircalabioru *et al*. [16] demonstrated that p22*^phox^* deficiency unexpectedly rescued mice from *C. rodentium* infection by harboring H_2_O_2_‐producing commensals. Therefore, it is critical to decipher how each NADPH oxidase subunit regulates intestinal inflammation.

Mutations in *NCF4*, the gene encoding p40*^phox^* subunit, have been shown to be associated with increased susceptibility for Crohn’s disease [17, 18, 19]. Conway *et al*. [9] reported that p40*^phox^* ^−/−^ mice showed increased susceptibility to both DSS‐ and anti‐CD40‐induced colitis. Ellson *et al*. [20] also demonstrated that p40*^phox^* ^−/−^ mice exhibited severe defects in ROS production in response to several stimuli. In that study, it was also shown that in the absence of p40*^phox^*, the expression of p67*^phox^* was reduced by 55% while the expression of p47*^phox^* was unaffected. Previously, we revealed that the p40*^phox^* subunit of NADPH oxidase played an essential role in the regulation of macrophage inflammatory response to *Salmonella* infection [21]. However, the role for p40*^phox^* in the regulation of intestinal immune response during infectious colitis is still unclear. Infection of mice with *Citrobacter rodentium*, an attaching‐effacing bacterium, has been widely used to study the potentially pathogenic host responses to enteric bacteria [22]. Therefore, using a model of *C. rodentium*‐induced colitis and genetically deficient mice, we intended to elucidate the impact of p40*^phox^* deficiency on host immune responses in the intestine and the development of colonic inflammation.

## Materials and methods

### Ethics approval

Animal studies were conducted in accordance with the recommendations in the Guide for the Care and Use of Laboratory Animals of the National Institutes of Health. The protocol was approved by the MGH Subcommittee on Research Animal Care (animal welfare assurance number A3596‐01).

### Mice

Six‐ to eight‐week‐old female C57BL/6 (wild‐type, B6) mice were obtained from the Jackson Laboratory (Bar Harbor, ME, USA). p40*^phox^* knockout (KO, p40*^phox^* ^−/−^) mice were kindly provided by R. Xavier (Massachusetts General Hospital, Boston, MA, USA). Generation of this p40*^phox^* ^−/−^ mice has been described in a previous study, and they have been backcrossed to the C57BL/6 background [20]. All mice were fed autoclaved food and water. Mice were maintained in a specific pathogen‐free facility at Massachusetts General Hospital (MGH) and were age‐ and gender‐matched for each experiment. Mice from the same experimental group were housed in different cages to control for co‐caging effects on the microbiota and host response to *C. rodentium* infection.

### *C*. *rodentium* infection

Mice (wild‐type and p40*^phox^* ^−/−^) were orally inoculated with *C. rodentium* strain DBS100 (ATCC, Manassas, VA, USA). Bacteria were grown overnight in Luria broth and resuspended in sterile PBS before infecting the mice (0.3 mL/mouse, 5 × 10^8^ CFU of *C. rodentium*). To evaluate the systemic effects of *C. rodentium* infection on the host, survival rates and body weight loss of the infected mice were measured throughout the experimental period.

### Quantitation of bacterial burden

Fecal pellets and selected organs were collected from *C. rodentium*‐infected mice (wild‐type and p40*^phox^* ^−/−^), weighed, and homogenized in sterile PBS. Serial dilutions of the homogenates were plated onto MacConkey agar plates. Bacterial colonies were quantified after overnight incubation at 37 °C.

### Histopathology

At necropsy, colonic tissues were isolated and frozen in Tissue‐Tek OCT compound (Miles Inc., Elkhart, IN, USA) and stored at −80 °C. Then, 5‐µm sections were cut and stained with hematoxylin and eosin. Pathology was scored using modified histological scoring systems previously described in literature [23]. Stained sections were analyzed without prior knowledge of the type of treatment.

### Quantitative real‐time PCR analysis

Total RNA was isolated from colon and spleen tissues using TRIzol reagent (Invitrogen Life Technologies, Carlsbad, CA, USA) following the manufacturer’s instruction. All RNA samples were reverse‐transcribed into cDNA using the Superscript First‐Strand Synthesis System (Invitrogen Life Technologies). The cDNA samples were then tested for the expression of interferon gamma (IFN‐γ), interleukin‐17 (IL‐17), IL‐10, IL‐6, tumor necrosis factor alpha (TNF‐α), IL‐1β, and inducible nitric oxide synthase (iNOS) by real‐time PCR performed as previously described [20]. Results were normalized with GAPDH mRNA levels, and relative quantification was calculated using the 2^−ΔΔCT^ method. The sequences of primers used to quantify mRNA are listed in Table 1.

**Table 1:** Primers used in this study.

Primer	Orientation	Sequence (5’‐3’)
GAPDH	F	TGGAATCCTGTGGCATCCATGAAAC
	R	TAAAACGCAGCTCAGTAACAGTCCG
IFN‐γ	F	TCAAGTGGCATAGATGTGGAAGAA
	R	TGGCTCTGCAGGATTTTCATG
IL‐17	F	CCACGTCACCCTGGACTCTC
	R	CTCCGCATTGACACAGCG
IL‐10	F	CCACAAAGCAGCCTTGCA
	R	AGTAAGAGCAGGCAGCATAGCA
IL‐6	F	GCTTAATTACACATGTTCTCTGGGAAA
	R	CAAGTGCATCATCGTTGTTCATAC
TNF‐α	F	CCCTCACACTCAGATCATCTTCT
	R	GCTACGACGTGGGCTACAG
IL‐1β	F	ACCTGTCCTGTGTAATGAAAGACG
	R	TGGGTATTGCTTGGGATCCA
iNOS	F	CAGAGGACCCAGAGACAAGA
	R	ACCTGATGTTGCCATTGTTG

### Immunofluorescence microscopy

To analyze the location and abundance of macrophages and neutrophils, colonic tissue sections were fixed in ice‐cold acetone, washed, and blocked with avidin/biotin agent (Vector Laboratories, Burlingame, CA, USA). Slides were then stained with FITC‐labeled anti‐mouse F4/80 (BD Pharmingen, San Diego, CA, USA) and Cy3‐labeled anti‐mouse Ly6G (BD Pharmingen). DNA was stained and mounted using the 4', 6‐diamidino‐2‐phenylindole (Vector Laboratories). All sections were analyzed by immunofluorescence microscopy (Nikon ECLIPSE 80i, Tokyo, Japan).

### Cytokine analysis

Mice (wild‐type and p40*^phox^* ^−/−^) were sacrificed by CO_2_ asphyxiation on day 7 postinfection. Mesenteric lymph node (MLN) lymphocytes and splenocytes were isolated as previously described [24]. Then, cells were seeded at a density of 5 × 10^6^ cells/mL in flat‐bottom 48‐well tissue culture plates precoated with 5 μg·mL^−1^ anti‐CD3 Abs (BD Pharmingen). After 72 h of incubation at 37 °C, culture supernatants were collected for subsequent cytokine analysis. Levels of IFN‐γ, IL‐17, IL‐10, and TNF‐α were measured using ELISA kits (BD Biosciences, San Jose, CA, USA) according to the manufacturer’s instruction. Cytokine production was calculated using mean values from triplicate cultures.

### Nitric Oxide (NO) Assay

Lymphocyte suspensions were prepared from spleens 7 days after infection. Cells (5 × 10^6^ cells/mL) were cultured in 48‐well plates in the presence or absence of LPS (100 ng·mL^−1^) or *C. rodentium* antigen (C‐Ag; 50 μg·mL^−1^). Culture supernatants were collected 48 h later for subsequent NO detection. *Citrobacter* antigen was prepared as previously described [25], and the concentrations of NO were measured by colorimetric assay kits (BioVision, Milpitas, CA, USA) following the manufacturer’s instructions.

### Statistical analysis

All data are expressed as means ± SEM. Statistical significance was determined using an unpaired Student’s *t*‐test or one‐way ANOVA followed by Tukey’s *post hoc* analysis with graphpad prism (GraphPad Software, San Diego, CA, USA). Significance was defined as a *P*‐value < 0.05.

## Results

### p40*^phox^* deficiency does not increase susceptibility of mice to *C. rodentium* infection

**Fig. 1:**
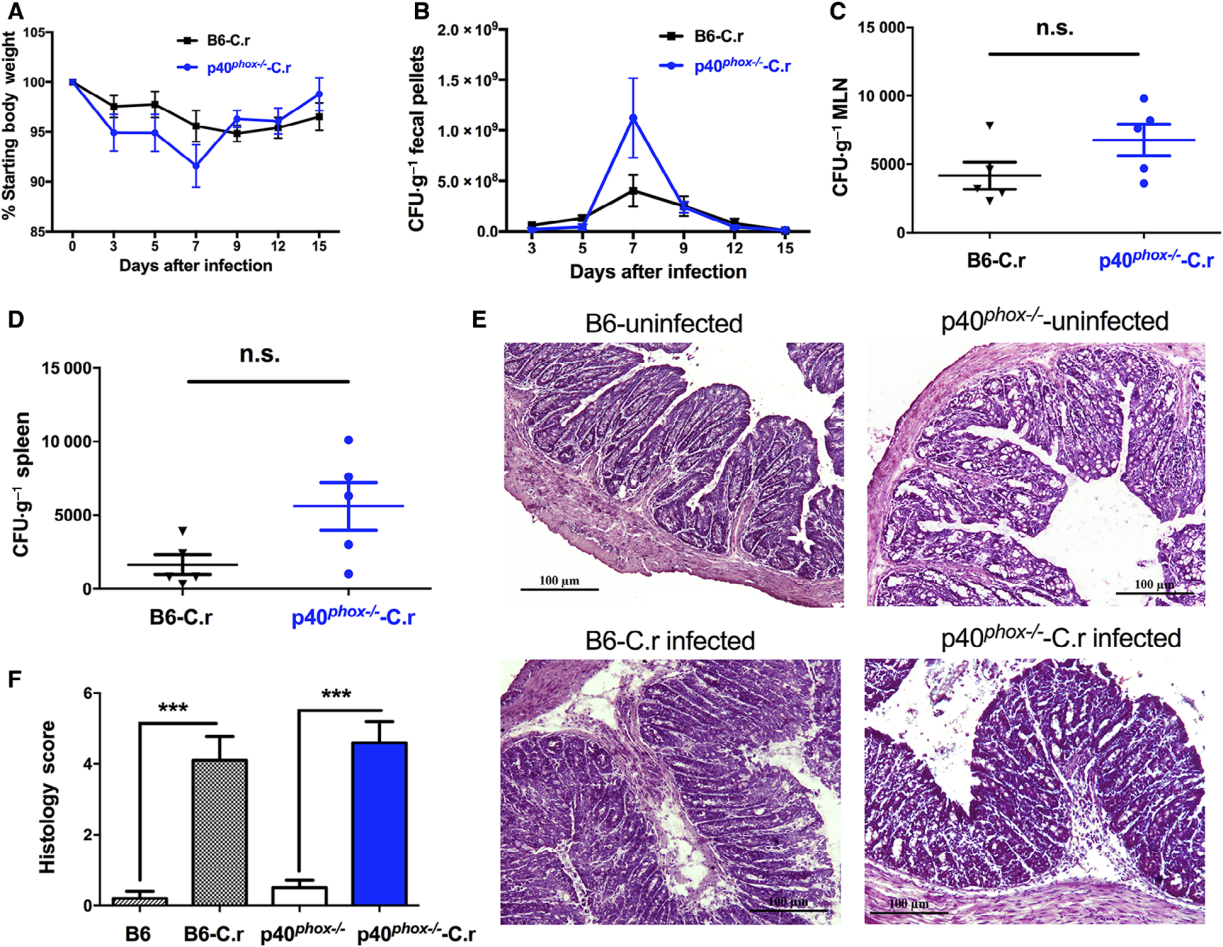
p40*^phox^* deficiency does not increase susceptibility of mice to *C. rodentium* infection. Wild‐type mice and mice lacking p40*^phox^* were infected orally with 5 × 10^8^ CFU of *C. rodentium* (DBS100 strain). Body weight changes of p40*^phox^* ^−/−^ and wild‐type mice were measured during the course of the experiment (A). Bacterial numbers in feces (B) and homogenates of MLN (C) and spleens (D) were determined. Significance was determined by Student’s *t*‐test. Data shown are mean ± SEM from one of three experiments performed showing similar results (*n* = 5/group; n.s., nonsignificant). At 7 days postinfection, colons were collected and processed for histological analysis. Representative H&E‐stained sections from uninfected B6 mice and p40*^phox^* ^−/−^ mice, B6 mice infected with *C. rodentium,* and p40*^phox^* ^−/−^ mice infected with *C. rodentium* are shown (original magnification ×100; scale bar, 100 μm) (E). Histopathological score of colonic inflammation in mice with *C. rodentium* infection (F). The scores were assessed by determination of infiltration of inflammatory cells (score range, 0 to 4), together with the evaluation of tissue damage (score range, 0–4). Statistical analysis was conducted using one‐way ANOVA followed by Tukey’s *post hoc* analysis. Data shown are mean ± SEM (*n* = 5/group; ***, *P* < 0.001).

To investigate the role of p40*^phox^* in host defense against *C. rodentium*, p40*^phox^* ^−/−^ mice and congenic C57BL/6 wild‐type mice were infected orally with 5 × 10^8^ CFU of *C. rodentium* (DBS100 strain). The course of bacterial infection in p40*^phox^*‐deficient mice was compared to that observed in wild‐type mice. Infection led to hunched posture, reduced activity, soft stool, and perianal fecal staining in both wild‐type and p40*^phox^* ^−/−^ mice. All mice survived over the course of *C. rodentium* infection. A similar degree and timing of body weight loss were found in both mouse strains during infection. As demonstrated in Fig. 1A, infected mice showed a gradual increase in their weight loss, reaching a maximum at 7 days after infection. Notably, no significant differences in maximal weight loss were observed between two groups. We next compared the fecal shedding of *C*. *rodentium* by plating serial dilutions on selective plates. Despite the trend toward increased fecal bacterial output in the p40*^phox^* knockout mice, it did not reach statistical significance (Fig. 1B). Consistently, analysis of the bacterial burden showed that both mouse strains had similar bacterial numbers present in the MLN (Fig. 1C) and spleens (Fig. 1D), although a trend of increase in bacterial load was noted in p40*^phox^* ^−/−^ mice 7 days after *C. rodentium* infection.

During the course of infection, colons of mice under basal and infected conditions were examined both macroscopically and microscopically. As expected, thicker colons were observed in infected mice (wild‐type and p40*^phox^* ^−/−^) 1 and 2 weeks after infection. However, no visible differences were observed between the groups (data not shown). At 7 days postinfection, histological examination revealed typical pathological changes in colons of infected wild‐type mice, including colonic crypt hyperplasia, submucosal edema, and inflammatory cell infiltrates, whereas these inflammatory features were absent from uninfected mice (Fig. 1E). In contrast to wild‐type mice, p40*^phox^*‐deficient mice infected with *C. rodentium* displayed pronounced disruption of epithelial architecture while less severe epithelial hyperplasia (Fig. 1E). Using the pathological scoring system [23], we found that the colonic inflammation scores for *C. rodentium*‐infected mice were significantly higher than those of uninfected control mice while there was no difference between wild‐type and p40*^phox^* ^−/−^ mice after infection (Fig. 1F).

### Differential cytokine expression in *C. rodentium*‐infected p40*^phox^*‐deficient mice

**Fig. 2:**
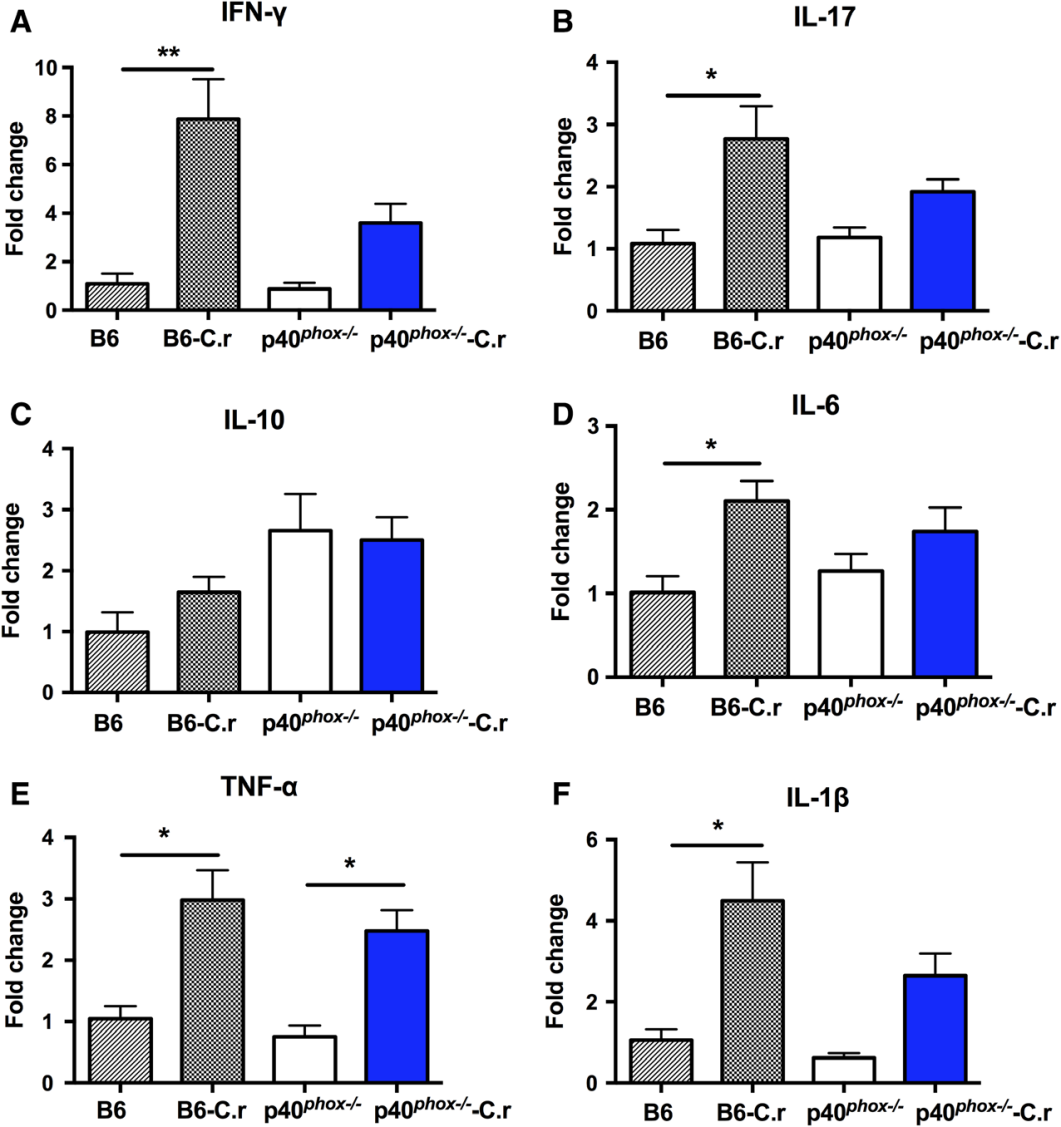
Colonic cytokine expressions in *C. rodentium*‐infected p40*^phox^*‐deficient mice. Colon tissues were collected for monitoring the cytokine gene expressions 7 days after infection. The expression levels of IFN‐γ (A), IL‐17 (B), IL‐10 (C), IL‐6 (D), TNF‐α (E), and IL‐1β (F) were detected by real‐time PCR. Statistical analysis was conducted using one‐way ANOVA followed by Tukey’s *post hoc* analysis. Values (mean ± SEM) represent the fold increase compared to baseline obtained from uninfected B6 mice (*n* = 5/group; *, *P* < 0.05; **, *P* < 0.01).

We then assessed the pro‐inflammatory cytokine profiles of colons from wild‐type and p40*^phox^* ^−/−^ mice infected with *C*. *rodentium* 7 days postinfection. Consistent with severe inflammatory changes in colonic tissues, *C. rodentium* infection significantly up‐regulated IFN‐γ (Fig. 2A), IL‐17 (Fig. 2B), IL‐6 (Fig. 2D), and IL‐1β (Fig. 2F) gene expression in infected wild‐type mice while there was no change in p40*^phox^* ^−/−^ mice. Notably, a higher expression level of IL‐10 (Fig. 2C) was observed in p40*^phox^* ^−/−^ mice (with or without infection) than that of wild‐type mice whereas the differences did not reach statistical significance. In addition, *C. rodentium* infection induced an up‐regulation of TNF‐α gene expression in colons of both mouse strains (Fig. 2E).

**Fig. 3:**
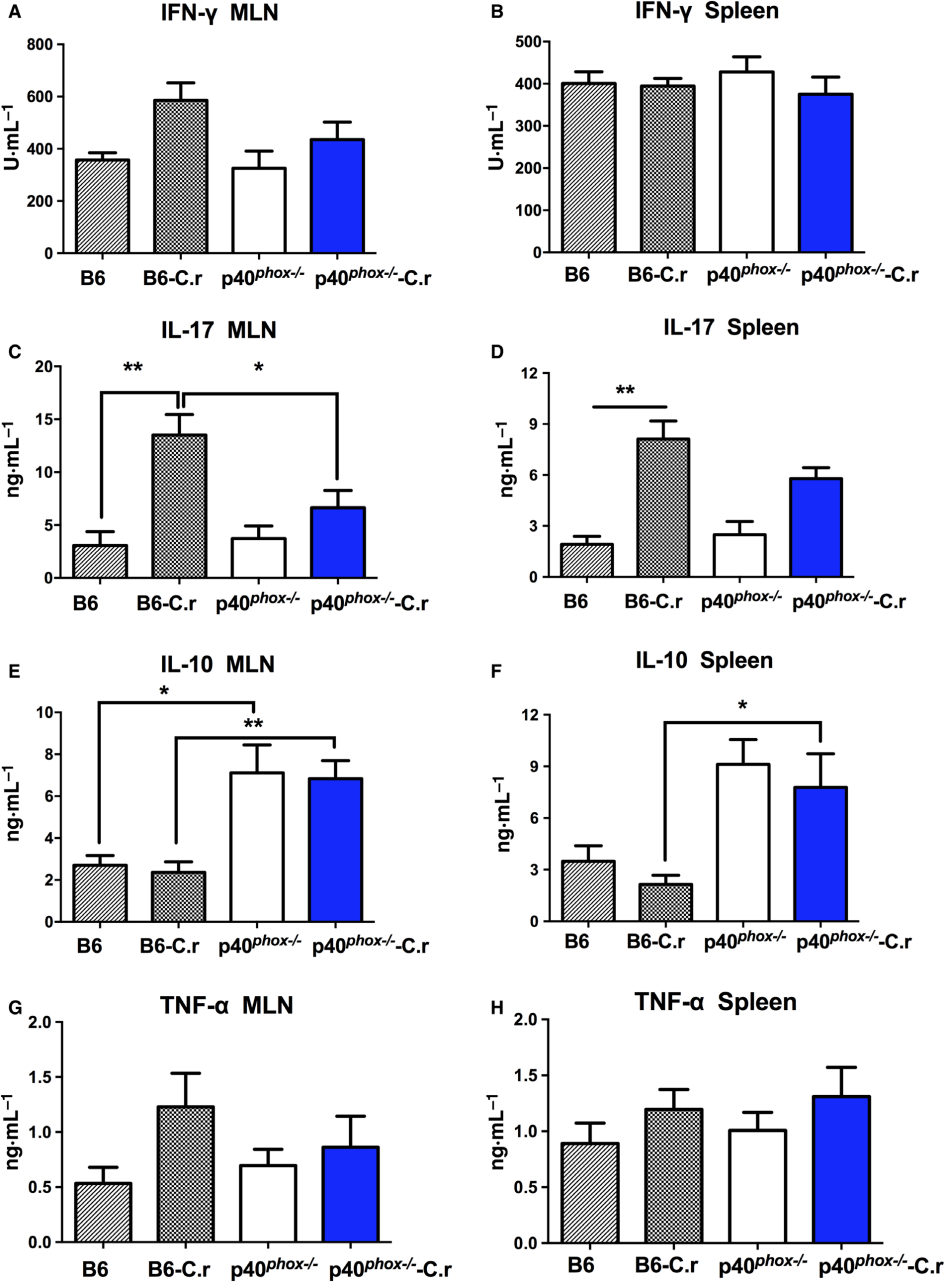
Altered cytokine production in MLN and spleens in p40*^phox^*‐deficient mice with *C. rodentium* infection. Cell culture supernatants of MLN lymphocytes and splenocytes were harvested and analyzed for cytokine production. Levels of IFN‐γ (A‐B), IL‐17 (C‐D), IL‐10 (E‐F), and TNF‐α (G‐H) were measured by ELISA techniques with commercially available kits. Cytokine production was calculated using mean values from triplicate cultures. One‐way ANOVA followed by Tukey's *post hoc* analysis was used. Data shown are mean ± SEM from one of three experiments performed showing similar results (*n* = 5/group; *, *P* < 0.05; **, *P* < 0.01).

Further ELISA analysis revealed that *C*. *rodentium* infection induced a Th1‐type response with increased IFN‐γ production in the culture supernatants of MLN cells isolated from wild‐type mice (Fig. 3A) whereas the differences did not reach statistical significance. No significant changes were found in IFN‐γ production in splenocytes after infection (Fig. 3B). Additionally, increased IL‐17 secretion in cell suspensions from MLN (Fig. 3C) and spleens (Fig. 3D) was detected after *C. rodentium* infection. In contrast, the levels of IL‐17 in MLN cell supernatants of infected p40*^phox^* ^−/−^ mice were significantly lower than those of infected wild‐type mice. However, IL‐10 levels in the supernatants of MLN (Fig. 3E) and spleens (Fig. 3F) cells of p40*^phox^* ^−/−^ mice under basal or infected conditions were higher than those in wild‐type mice. Further, there was no difference in the levels of TNF‐α in MLN (Fig. 3G) and spleens (Fig. 3H) post‐*C*. *rodentium* infection.

### Inflammatory cell infiltration of the colonic mucosa with *C. rodentium* infection

**Fig. 4:**
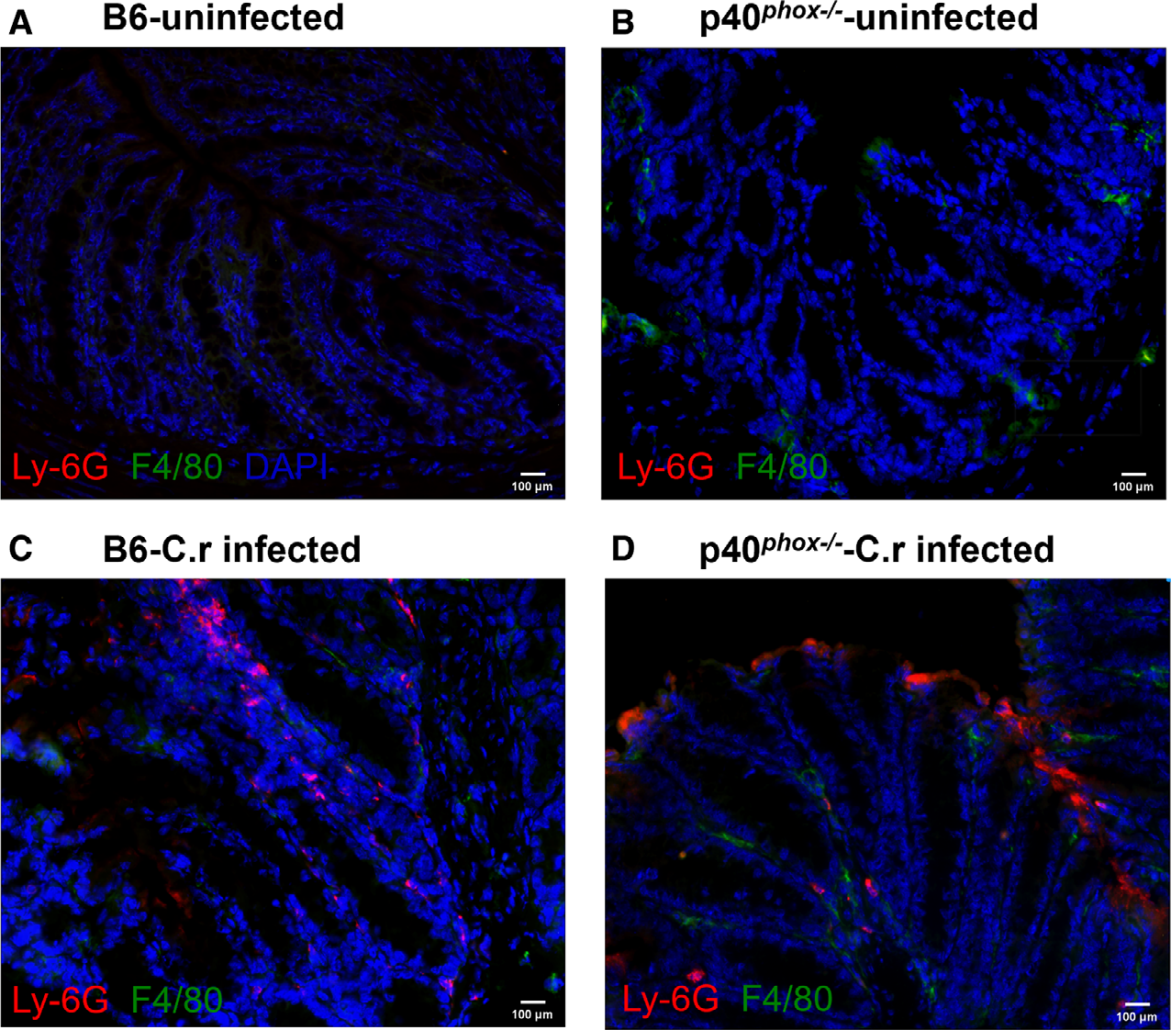
Inflammatory cell infiltration of the colonic mucosa with *C. rodentium* infection. Mice deficient in p40*^phox^* and normal B6 mice were infected with *C*. *rodentium* and sacrificed 7 days postinfection. Colon sections from uninfected B6 (A) and p40*^phox^* ^−/−^ (B) mice and infected B6 (C) and p40*^phox^* ^−/−^ (D) mice were stained with anti‐F4/80 (green) for macrophages and anti‐Ly6G (red) for neutrophils (magnification ×200; scale bar, 100 μm).

To characterize the infiltrating cell population in infected mice (wild‐type and p40*^phox^* ^−/−^), colonic sections were stained with anti‐F4/80 (green) for macrophages and anti‐Ly6G (red) for neutrophils. As shown in Fig. 4C,D, a clear infiltration of F4/80^+^ macrophages and Ly6G^+^ neutrophils was noted in both mouse strains after infection, compared with mice without infection (Fig. 4A,B).

### p40*^phox^* deficiency increases iNOS expression and NO production in response to *C. rodentium* infection

**Fig. 5:**
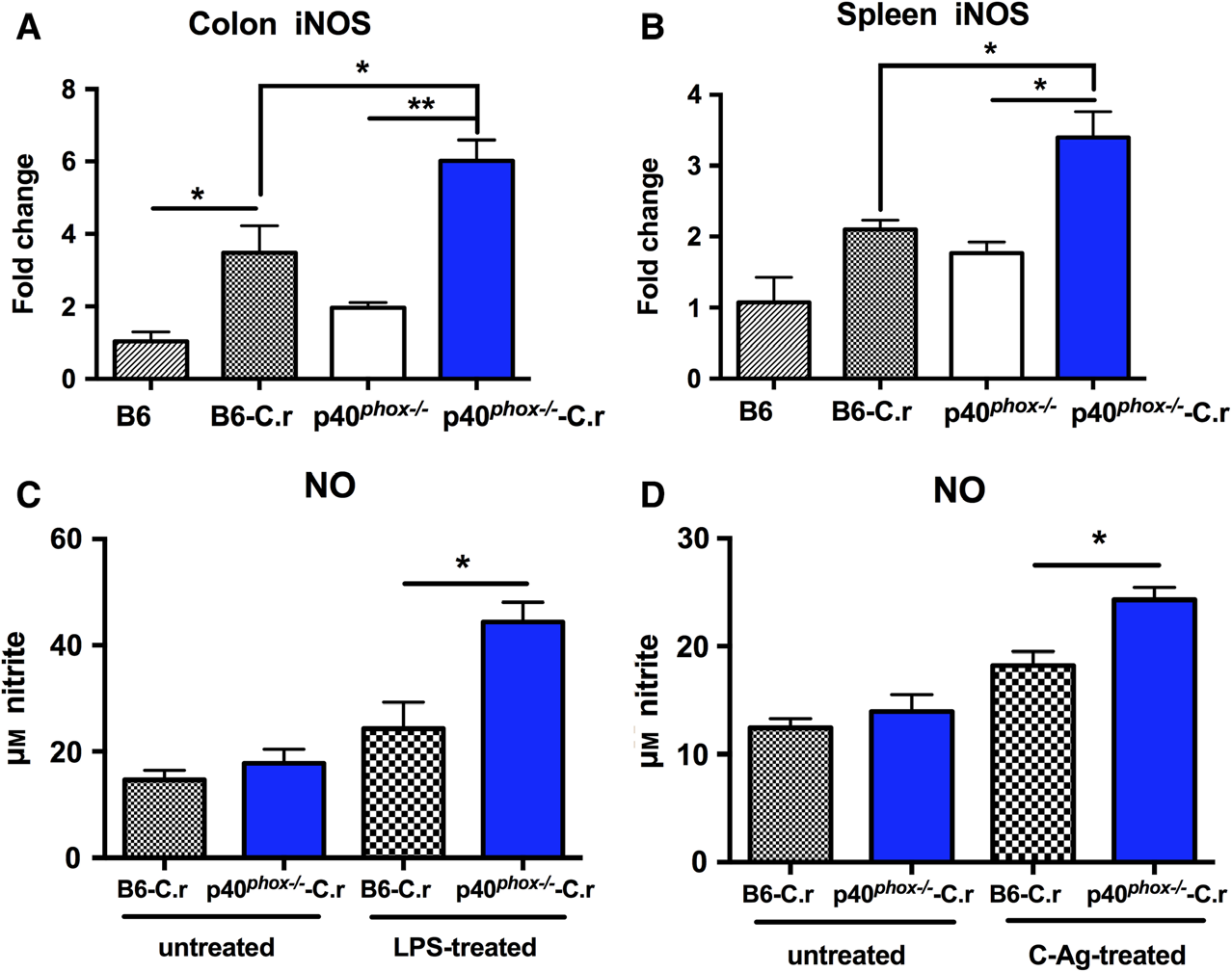
p40*^phox^* deficiency increases iNOS expression and NO production in response to *C. rodentium* infection. Colon (A) and spleen (B) tissues were collected to determine iNOS expression profile 7 days after *C. rodentium* infection. Statistical analysis was conducted using one‐way ANOVA followed by Tukey’s *post hoc* analysis. NO concentrations in the culture supernatants of splenocytes after exposure to LPS (C) or C‐Ag (D) were measured by colorimetric assay kits following the manufacturer’s instructions. Significance was determined by Student’s *t*‐test. Data shown are mean ± SEM from one of three experiments performed showing similar results (*n* = 5/group; *, *P* < 0.05; **, *P* < 0.01).

We next examined the iNOS expression profile and NO secretion in the two mouse strains under basal or infected conditions. As depicted in Fig. 5A,B, *C*. *rodentium* infection induced a remarkable up‐regulation of iNOS mRNA expression in both colon and spleen tissues from p40*^phox^* ^−/−^ mice compared with wild‐type mice. Based on the increased iNOS expression, we examined the production of NO in splenocytes culture supernatants after *in vitro* exposure to LPS or C‐Ag. As illustrated in Fig. 5C,D, NO levels were significantly greater in infected mice lacking p40*^phox^* than those in wild‐type mice with LPS or bacterial antigen re‐stimulation. Collectively, these data suggest that defects in NADPH oxidase may result in enhanced iNOS expression and NO production in response to *C. rodentium* infection.

## Discussion

The NADPH oxidase complex is critical for bacterial killing [26, 27] while its deficiency may lead to increased susceptibility to infection and inflammatory disease [28]. Up to date, mixed results have been reported. Conway *et al*. [9] demonstrated that p40*^phox^*‐deficient mice had increased susceptibility to DSS‐induced colitis. However, other investigators showed that gp91*^phox^*‐ and p47*^phox^*‐deficient mice did not exhibit enhanced inflammation after DSS treatment [14, 15]. In the current study, the *C. rodentium* infection model, a robust model of infectious disease and colitis, was used and we found that both mouse strains (wild‐type and p40*^phox^* ^−/−^) exhibited a similar degree and timing of body weight loss during the course of *C. rodentium* infection. Consistent with previous studies [29, 30], high fecal shedding and bacterial burden were observed by day 7 after *C. rodentium* infection. Despite the trend toward increased fecal bacterial output and tissue bacterial loads in mice lacking p40*^phox^*, it did not reach statistical significance. The hallmark pathological feature of *C. rodentium* infection is colonic crypt hyperplasia [31, 32]. We demonstrated here that colonic inflammation developed to a similar extent in both mouse strains (wild‐type and p40*^phox^* ^−/−^) infected with *C. rodentium*. Our results suggest that p40*^phox^* deficiency does not increase susceptibility of mice to *C. rodentium* infection. Similar results were obtained by Fattouh *et al*. [5], who demonstrated that gp91*^phox^* ^−/−^ mice infected with *C. rodentium* did not develop exaggerated colonic hyperplasia or colitis. Interestingly, Pircalabioru *et al*. [16] showed that p22*^phox^* deficiency unexpectedly rescued mice from *C. rodentium* infection, by fostering H_2_O_2_‐producing gut commensals, which physically displaced and attenuated *C. rodentium* virulence. In another study, Falcone *et al*. [33] reported that p47*^phox^* deficiency increased susceptibility to DSS colitis, while breeding p47*^phox+/−^* mice and standardizing the microflora between littermate p47*^phox^* ^−/−^ and wild‐type mice from birth, significantly reduced DSS and *C. rodentium* colitis susceptibility in p47*^phox^* ^−/−^ mice. These published data indicate the complexity of the relationship among host produced ROS, gut microbiota, enteric pathogens, and the pathogenesis of IBD, and further studies involving intestinal microbiome analysis are needed to clarify the role for p40*^phox^* in enteric disease.

Previous studies have shown that oral infection with *C. rodentium* elicits a robust immune response characterized by a mixed Th1/Th17 response in intestinal mucosa, contributing to both host defense and tissue damage [30, 34]. In agreement with these studies, we found that mice infected with *C. rodentium* had increased gene expression of multiple pro‐inflammatory cytokines, including IFN‐γ, IL‐17, IL‐6, TNF‐α, and IL‐1β. The increased expression of inflammatory cytokines was consistent with the severe inflammatory changes observed in colonic tissues and further supported by the results from our immunofluorescence microscopic analysis revealing enhanced infiltration of macrophages and neutrophils at mucosal sites after infection. Noticeably, a trend toward enhanced IL‐10 expression and decreased Th17 response was noted in infected mice deficient in p40*^phox^*. We further evaluated the T‐cell responses in lymphocytes collected from MLN and spleens, following re‐stimulation with anti‐CD3 MAb. In line with previous studies [25, 35], we demonstrated that *C. rodentium* infection resulted in a robust Th1‐ and Th17‐type response with increased IFN‐γ and IL‐17 production in wild‐type MLN cells. Notably, a weak production of Th1/Th17‐related cytokines was observed in infected p40*^phox^*‐deficient mice. Compared with wild‐type mice, higher IL‐10 levels in the supernatants of MLN and spleen cells of p40*^phox^* ^−/−^ mice were noted. Chaudhry *et al*. [36] described that anti‐inflammatory IL‐10 could endow Treg cells with the ability to suppress pathogenic Th17 cell responses. Therefore, the observed impaired Th17 responses may be associated with the high levels of IL‐10 present in p40*^phox^* ^−/−^ mice.

Evidence has shown that iNOS‐derived NO, a highly reactive molecule, plays an integral role in host innate immunity against various infections [37, 38, 39]. While the antimicrobial activity of NO against intracellular bacteria is well known, its role in defending against extracellular mucosal pathogens is less clear [40]. Previous studies have reported that *C. rodentium* infection could induce iNOS expression and the subsequent release of NO in the inflamed colons [41, 42]. Moreover, Vallance *et al*. [41] revealed that colonic epithelial cells rather than phagocytes were the main cellular source of iNOS, directly induced by bacterial attachment. In accordance with these findings, our results demonstrated that iNOS mRNA expression in the colon and spleen tissues significantly increased after *C. rodentium* infection. Consistently, NO levels were significantly greater in p40*^phox^*‐deficient mice than those in wild‐type mice with LPS or bacterial antigen re‐stimulation. Niedbala *et al*. [43] reported that NO was able to induce the proliferation of immunosuppressive Treg cells, and those NO‐Tregs could attenuate colitis in an IL‐10‐dependent manner. In keeping with this view, the increased NO synthesis might result in increased IL‐10 production observed in p40*^phox^*‐deficient mice. Therefore, our observations indicate that defects in p40*^phox^* may enhance iNOS expression and NO production, which may lead to alterations in IL‐10 production and colitis development.

In summary, our findings demonstrate that p40*^phox^*‐deficient mice subjected to an infection‐based model of colitis do not develop worsened disease, since no differences were found in body weight loss, bacterial clearance, colonic pathology, cytokine production, or immune cell recruitment between two mouse strains. The mechanisms are presently unclear, but one could speculate that altered cytokine responses and enhanced iNOS and NO levels may be involved. Our study provides a further understanding of the complex interaction between NADPH oxidase, immune defense, and enteric pathogens, which may facilitate the development of therapeutic strategies for intestinal disease.

## Conflict of interest

The authors declare no conflict of interest.

## Author contributions

YY, YL, and ML performed the experimental work, acquired the data, analyzed, and interpreted results. YY and YL wrote the first draft of the manuscript. WL and HS conceived and designed the project, critically revised the manuscript. All authors read and approved the final manuscript.

## Data Availability

All data will be available from the corresponding author upon reasonable request.
